# Glutathione overproduction mediates lymphoma initiating cells survival and has a sex-dependent effect on lymphomagenesis

**DOI:** 10.1038/s41419-024-06923-z

**Published:** 2024-07-27

**Authors:** Alberto H.-Alcántara, Omar Kourani, Ana Marcos-Jiménez, Patricia Martínez-Núñez, Estela Herranz-Martín, Patricia Fuentes, María L. Toribio, Cecilia Muñoz-Calleja, Teresa Iglesias, Miguel R. Campanero

**Affiliations:** 1 Cell-cell communication and inflammation Unit, Centro de Biología Molecular Severo Ochoa (CBM), CSIC-UAM, Madrid, Spain; 2 Immunology Department, Instituto de Investigación Sanitaria Princesa, Hospital Universitario de la Princesa, Madrid, Spain; 3 Immune System Development and Function Unit, CBM, CSIC-UAM, Madrid, Spain; 4 Centro de Investigación Biomédica en Red en Enfermedades Infecciosas (CIBERINFEC) Madrid, Madrid, Spain; 5 Facultad de Medicina, Universidad Autónoma de Madrid, Madrid, Spain; 6 Department of Neurological Diseases and Aging, Instituto de Investigaciones Biomédicas Sols-Morreale, CSIC-UAM, Madrid, Spain; 7 Centro de Investigación Biomédica en Red de Enfermedades Neurodegenerativas (CIBERNED) Madrid, Madrid, Spain; 8 Centro de Investigación Biomédica en Red en Enfermedades Cardiovasculares (CIBERCV), Madrid, Spain

**Keywords:** B-cell lymphoma, Acute lymphocytic leukaemia, Chronic lymphocytic leukaemia, Apoptosis

## Abstract

Lymphoid tumor patients often exhibit resistance to standard therapies or experience relapse post-remission. Relapse is driven by Tumor Initiating Cells (TICs), a subset of tumor cells capable of regrowing the tumor and highly resistant to therapy. Growing cells in 3D gels is a method to discern tumorigenic cells because it strongly correlates with tumorigenicity. The finding that TICs, rather than differentiated tumor cells, grow in 3D gels offers a unique opportunity to unveil TIC-specific signaling pathways and therapeutic targets common to various cancer types. Here, we show that culturing lymphoid cells in 3D gels triggers reactive oxygen species (ROS) production, leading to non-tumor lymphoid cell death while enabling the survival and proliferation of a subset of lymphoma/leukemia cells, TICs or TIC-like cells. Treatment with the antioxidant N-acetylcysteine inhibits this lethality and promotes the growth of primary non-tumor lymphoid cells in 3D gels. A subset of lymphoma cells, characterized by an increased abundance of the antioxidant glutathione, escape ROS-induced lethality, a response not seen in non-tumor cells. Reducing glutathione production in lymphoma cells, either through pharmacological inhibition of glutamate cysteine ligase (GCL), the enzyme catalyzing the rate-limiting step in glutathione biosynthesis, or via knockdown of *GCLC*, the GCL catalytic subunit, sharply decreased cell growth in 3D gels and xenografts. Tumor cells from B-cell lymphoma/leukemia patients and λ-MYC mice, a B-cell lymphoma mouse model, overproduce glutathione. Importantly, pharmacological GCL inhibition hindered lymphoma growth in female λ-MYC mice, suggesting that this treatment holds promise as a therapeutic strategy for female lymphoma/leukemia patients.

## Introduction

Many conventional cancer therapies often lead to undesired side effects because they target proteins and cellular processes necessary for normal cell survival and proliferation, including various progenitor cell types. Identifying genes and proteins specifically associated with tumor traits would facilitate the development of therapies that selectively target tumor cells while minimizing toxic effects. Despite some potentially fatal side effects, the major failures of current cancer treatments are the lack of response in certain human cancer types and tumor relapse after initial remission [1].

Despite progress in cancer research and the development of new potential therapies, >90% of cancer treatments exhibiting preclinical activity fail in human trials [2, 3]. This failure is largely due to testing exclusively on cancer cell lines grown in liquid medium, a condition that predicts in vivo efficacy to anticancer drugs worse than cell culture in 3D gels [4, 5] and is far from adequately reflecting the therapeutic responsiveness of cancers within their native microenvironment [2, 3]. Unfortunately, one-third of cancer patients succumb to the disease within five years of diagnosis, primarily due to tumor recurrence [1, 6]. Discovering strategies to prevent tumor relapse continues to be a challenge.

A major challenge in developing effective anti-cancer therapies is identifying tumor-specific properties. One such property is the altered redox state, which is observed in many forms of cancer [7]. Low levels of reactive oxygen species (ROS) mediate signaling cascades involved in cell proliferation [8], while high ROS levels can lead to DNA damage and eventual cell death [9]. To neutralize ROS, cells induce various antioxidant systems, including the activation of the protein machinery that produces glutathione [10], the most abundant antioxidant scavenger in all cells [11, 12]. Glutathione is synthesized in two sequential enzymatic reactions: glutamate cysteine ligase (GCL) catalyzes the first, rate-limiting step, and glutathione synthetase couples γ-glutamylcysteine to glycine in the last step [13]. GCL comprises a catalytic (GCLC) and a modifier (GCLM) subunit, and its activity can be readily inhibited with DL-buthionine-sulphoximine (BSO) [13]. Additionally, a family of enzymes expressed on the outer membrane of cells, gamma-glutamyl transpeptidases (GGTs), is involved in recycling extracellular glutathione, thereby contributing to de novo intracellular glutathione biosynthesis and ROS detoxification [14].

Growing evidence suggests that numerous cancers, including hematopoietic tumors, may be driven by a small subset of cells known as tumor-initiating cells (TICs) [15]. These TICs exhibit unique properties such as self-renewal and high tumorigenicity, making them the only cancer cells capable of regenerating the original tumor [16]. Since these cells are the most resistant cells to chemotherapy and radiotherapy within the tumor [17], they are considered as responsible for tumor relapse [15]. Their resistance has been linked to their capacity to reduce ROS levels through the accumulation of intracellular antioxidants like glutathione and the induction of GCL, as demonstrated in breast and liver TICs [18–20].

While the discovery of therapeutic strategies targeting TICs holds promise, our understanding of TIC biology remains limited due to the challenges in identifying and isolating them. Conventional cell surface markers are often unreliable and vary among cancer types [21, 22]. However, a breakthrough came with the observation that TICs, rather than the bulk of differentiated tumor cells, thrive in 3D gels [23, 24], offering an opportunity to uncover signaling pathways specific to TIC growth and identify TIC-specific therapeutic targets.

Growth in 3D gels strongly correlates with tumorigenicity [25], and this property is used to distinguish tumorigenic from non-tumorigenic cells in vitro. Carcinoma cells, for instance, form colonies in 3D gels, whereas immortalized NIH-3T3 fibroblasts do not [26, 27]. Similarly, lymphoblastoid B-cell lines (LCLs), non-tumor cell lines created through the immortalization of normal B-cells from healthy donors using the Epstein-Barr virus (EBV), do not grow in 3D gels [28, 29]. In contrast, a small fraction of lymphoma and leukemia cell lines successfully grow in these gels [28, 30], suggesting the presence of TIC or TIC-like cells even in established lymphoma/leukemia cell lines cultured in vitro. These findings align with previous reports indicating the maintenance of stem-cell-like cancer cells in established carcinoma cell lines [31].

It has been proposed that epithelial and mesenchymal cells survival relies on the signaling provided by integrins attached to a rigid surface and that culturing them in 3D gels leads to cell death by preventing attachment to a rigid surface [26]. Importantly, gels not only prevent anchorage to a rigid surface but also to other cells. It should be noted that while lymphoid cells remain viable in the circulatory system without anchoring to a rigid surface, they do not proliferate there. Instead, they proliferate in lymphoid organs, where interactions with extracellular matrix, stromal cells, and/or other lymphocytes may play a role.

Despite the strong correlation between growth within 3D gels and tumorigenicity [25], it remains unclear why TICs are the exclusive tumor cells capable of growing in this condition, while non-tumor lymphoid cells do not. Investigating the molecular mechanisms enabling TIC growth within 3D gels could unveil new targets to selectively inhibit TIC growth and prevent lymphomagenesis. To address this, we have established experimental conditions for retrieving cells from a 3D gel and analyzing them by flow cytometry. These studies indicate that overproduction of glutathione drives the survival and growth of a small subset of lymphoid tumor cells in 3D gels and xenografts, likely TIC or TIC-like cells, and that glutathione is selectively required for tumor growth in females of a mouse model of lymphoma.

## Results

### 3D gels selectively support the survival of a subset of lymphoid tumor cells

**Fig. 1:**
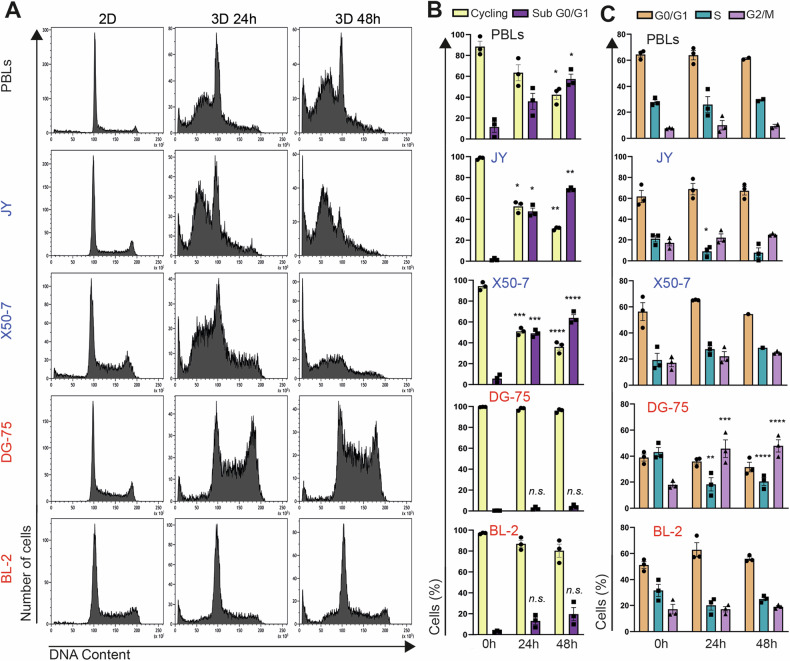
Cell death limits growth of lymphoid cells within a 3D gel. **A** Representative cell cycle profiles, (**B**) quantification of cycling cells (cells in G0/G1, S and G2/M) and apoptotic cells (SubG0/G1), and (**C**) quantification of cells in either G0/G1, S, or G2/M phase of the cell cycle in PBLs, LCLs (JY and X50-7), and lymphoma B-cells (DG-75 and BL-2) grown in 2D liquid culture (2D) or in 3D gels (3D) for 24 h or 48 h. **B**, **C** Each data point denotes the value from an independent experiment and data in histograms are presented as mean ± s.e.m. *p < 0.05, **p < 0.01, ***p < 0.001, ****p < 0.0001 vs 0 h; n.s., non-significant; two-way ANOVA with Bonferroni post hoc test.

While growth rates of LCLs (X50-7 and JY) and lymphoma B cells (DG-75 and BL-2) in 2D culture conditions are nearly identical [28], lymphoma B cells form colonies within 3D gels, unlike LCLs (Fig. S1A, B). These results are consistent with previous findings [28, 30, 32]. Notably, RT-qPCR analysis showed a sharp increase in *OCT4* mRNA expression, a marker of pluripotent stem cells and essential for TIC self-renewal [33], in the colonies formed in 3D gels relative to 2D culture conditions (Fig. S1C). This supports the notion that only TICs or TIC-like cells grow in soft gels. PBLs require proper stimulation and IL-2 for proliferation in 2D culture conditions [34]. To understand why non-tumor lymphoid cells do not grow within 3D gels, we cultured activated PBLs, LCLs, and lymphoma cells within such gels for 24–72 h and then analyzed their cell cycle and viability using flow cytometry. We established the experimental conditions to recover cells embedded in the hydrogel and stain them with propidium iodide. Within just 24 h in the gel, 40–50% of PBLs, JY, and X50-7 non-tumor cells underwent apoptosis, as indicated by an increase in the sub-G_0_/G_1_ population, and this effect was exacerbated after 48 h (Fig. 1A, B). In contrast, <20% of lymphoma cells (DG-75 and BL-2) entered apoptosis after 24 h (Fig. 1A, B). While DG-75 cells modestly accumulated in the G_2_/M phase of the cell cycle, JY exhibited a slight decrease in the S phase, and the cell-cycle distribution of PBLs and X50-7 remained largely unaffected (Fig. 1C).

Flow cytometry analysis of cell size (FSC) and granularity (SSC) detected changes in cell viability (Fig. S2), revealing a substantial decrease in viability of JY, X50-7, and BL-2 cells after 24 h of culture within a 3D gel, with a milder reduction in DG-75 cells viability (Fig. S3). The decline in viability among B-cell lymphomas was accentuated after 48 h, and this effect was also observed in the Jurkat acute T-cell leukemia cell line (Fig. S3). Similar results were obtained when analyzing cells stained with calcein-AM, a viability marker (Fig. S4). Together, these findings strongly suggest that the limited growth of most lymphoid cells in 3D gels primarily results from cell death, rather than cell-cycle arrest, while a small subset of lymphoid tumor cells, likely TIC or TIC-like cells, maintain viability within the gel.

### Oxidative stress induces cell death in 3D gels

**Fig. 2:**
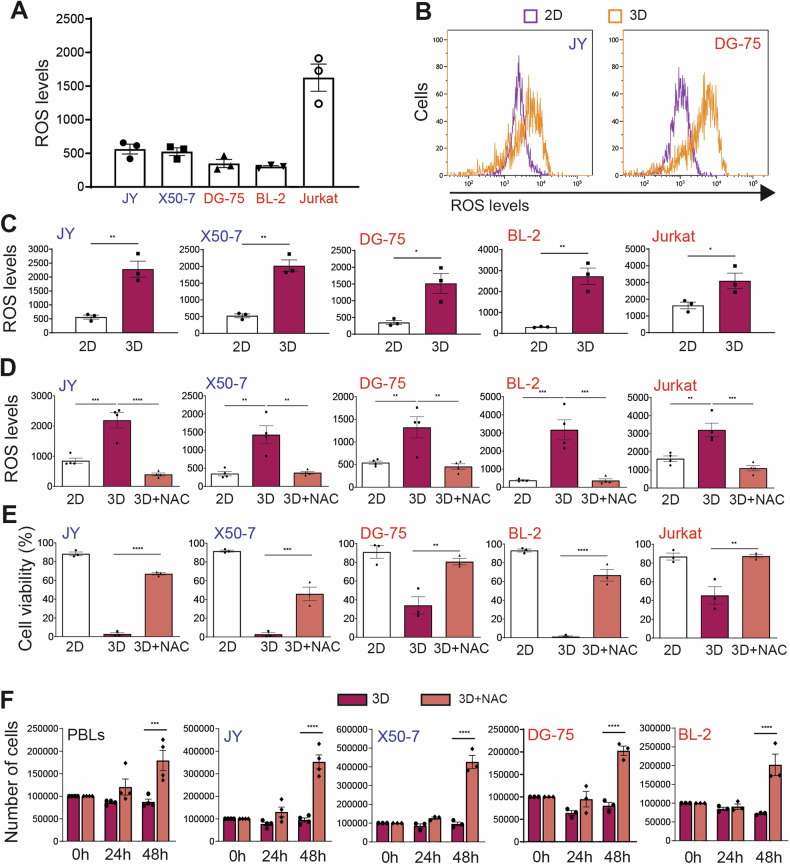
Increased oxidative stress mediates the lethality induced by cell culture within a 3D gel. Flow cytometry analysis of ROS staining with DCFDA in the indicated cells cultured in (**A**) 2D liquid medium (2D); (**B**, **C**) 2D or 3D gels (3D) for 24 h; and (**D**) 2D and 3D for 72 h in the presence of 5 mM N-acetylcysteine (NAC), as indicated. **(E)** Flow cytometry analysis of calcein staining in the indicated cells cultured either in 2D or 3D for 72 h. **A**, **C**, **D**, **E** Each data point denotes the value from an independent experiment and data in histograms are presented as mean ± s.e.m. **C** *p < 0.05, **p < 0.01; Student t-test; (**D**, **E**) **p < 0.01, ***p < 0.001, ****p < 0.0001; one-way ANOVA with Bonferroni post hoc test. **F** Primary normal peripheral blood lymphocytes (PBLs) and JY, X50-7, DG-75, and BL-2 cells (10^5^ cells) were seeded within 3D gels in the absence (3D) or presence of 5 mM NAC (3D + NAC) and recovered immediately after jellification (0) or after 24 h and 48 h of culture within the gel. Each data point denotes the number of cells recovered in an independent experiment and data in histograms are presented as mean ± s.e.m. ***p < 0.001, ****p < 0.0001; two-way ANOVA with Bonferroni post hoc test.

Given that oxidative stress can lead to cell death [9], we investigated whether cell culture within a 3D gel increased ROS generation. Using flow cytometry, we analyzed cells stained with 2’,7’–dichlorofluorescein diacetate (DCFDA), a fluorescent dye for ROS detection. In 2D culture conditions, LCLs and lymphoma cells showed similar intracellular ROS levels, with higher ROS in Jurkat cells (Fig. 2A).

However, after 24 h within a 3D gel, all cell types exhibited a sharp rise in ROS levels (Fig. 2B, C). Addition of N-acetylcysteine (NAC), a potent antioxidant, substantially decreased ROS levels in cells cultured for 72 h within these gels (Fig. 2D), improved the viability of lymphoid tumor cells and LCLs in the hydrogel (Fig. 2E) and promoted rapid growth within 3D gels for these cell types and even PBLs from healthy donors (Fig. 2F).

### Glutathione is required for lymphoid tumor cell growth in 3D gels and xenografts

**Fig. 3:**
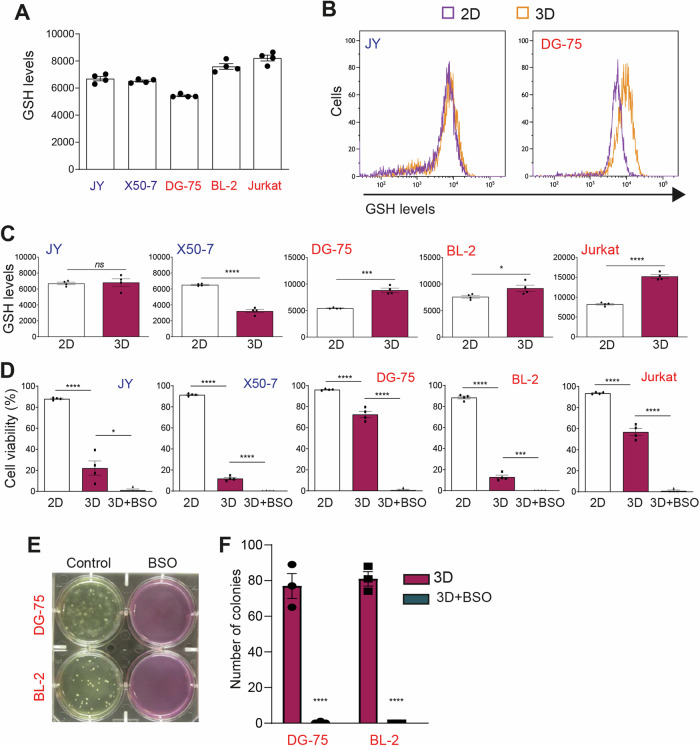
Glutathione overproduction mediates survival and growth of TIC within 3D gels. Flow cytometry analysis of glutathione (GSH) staining with mBcl in the indicated cells cultured in (**A**) liquid medium (2D) and (**B**, **C**) 2D or 3D gels (3D) for 24 h. (**D**) Rate of living cells cultured 24 h in 2D, 3D, or 3D supplemented with the GCL inhibitor BSO (50 µM), as determined by flow cytometry analysis of cell size and granularity. **E** Images of representative wells containing the indicated cells grown in 3D or 3D supplemented with 50 µM BSO for 21 days and (**F**) number of colonies per well. **A**, **C**, **D**, **F** Each data point denotes the value from an independent experiment and data in histograms are presented as mean ± s.e.m. **C** *p < 0.05, ***p < 0.001, ****p < 0.0001, n.s., non-significant vs 2D, Student t-test. **D** *p < 0.05, ***p < 0.001, ****p < 0.0001; one^-^way ANOVA with Bonferroni post hoc test. **F** ****p < 0.0001 vs 3D, two-way ANOVA with Bonferroni post hoc test.

To investigate whether the survival of TIC-like cells within 3D gels is mediated by ROS neutralization with glutathione, the most abundant antioxidant scavenger in all cells [11, 12], we assessed intracellular glutathione levels in lymphoid tumor cells and LCLs. Flow cytometry analysis of cells stained with mBcl, a fluorescent dye for glutathione detection, revealed similar intracellular glutathione levels in LCLs and lymphoma/leukemia cells in 2D culture conditions (Fig. 3A). However, when cultured within 3D gels for 24 h, lymphoid tumor cells exhibited increased levels of reduced glutathione, while levels remained unchanged or decreased in LCLs (Fig. 3B, C).

The increase in reduced glutathione may be achieved through increased glutathione reductase activity, elevated NADPH levels, or enhanced GCL-mediated glutathione biosynthesis [13]. To investigate whether increased glutathione biosynthesis accounts for TIC-like survival within a 3D gel, we treated lymphoid tumor cells with DL-Buthionine-sulphoximine (BSO), a pharmacological inhibitor of GCL-mediated glutathione biosynthesis. This treatment markedly decreased glutathione levels (Fig. S5A) and increased ROS accumulation (Fig. S5B) in LCLs and lymphoid tumor cells in 2D liquid culture without substantially decreasing their viability (Fig. S5C). In contrast, BSO treatment blunted the viability of lymphoid tumor cells within 3D gels (Fig. 3D) and blocked colony formation (Fig. 3E, F).

**Fig. 4:**
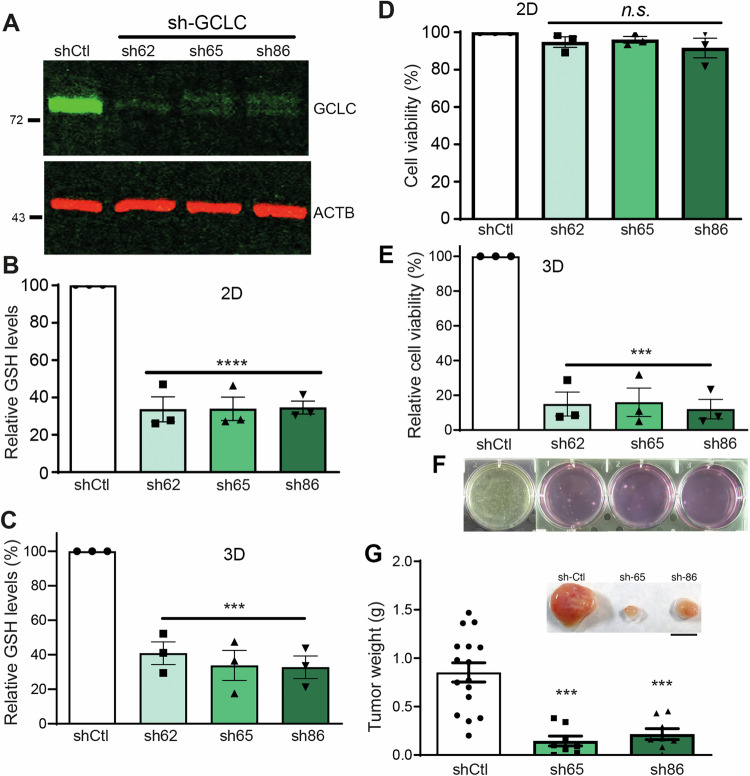
GCLC silencing impairs lymphoma cells survival and growth within 3D gels and lymphomagenesis in vivo. DG-75 cells were transduced with lentivirus encoding a puromycin-inactivating protein and either a control shRNA (shCtl) or the *GCLC*-specific shRNAs sh-62, sh-65, or sh-86. Transduced cells were selected by culture for >5 days in liquid medium containing 2 μg/ml puromycin and then further cultured either in liquid medium (2D) or within 3D gels (3D). **A** Immunoblot analysis for GCLC and ACTB (loading control) expression in protein extracts from DG-75 cells transduced with lentivirus expressing the indicated shRNAs. Uncropped images of these immunoblots are shown in a Supplementary File. Flow cytometry analysis of the staining of the indicated transduced cells with (**B**, **C**) mBcl or (**D**, **E**) calcein after 24 h of culture in 2D (**B**, **D**) or 3D (**C**, **E**). Each data point denotes the value from an independent experiment and data in histograms are presented as mean ± s.e.m. ***p < 0.001, ****p < 0.0001; one-way ANOVA with Bonferroni post hoc test. **F** Images of representative wells containing the indicated transduced DG-75 cells grown within 3D gels for 21 days. **G** Image of representative tumors and weight of tumors in NOD-SCID mice subcutaneously inoculated with DG-75 cells transduced with shCtl (*n* = 16 mice), sh-65 (*n* = 8 mice), or sh-86 (*n* = 8 mice). Each data point denotes the tumor of an individual mouse, and the horizontal bars denote the mean (long bar) and s.e.m.; ***p < 0.001 vs sh-Ctl; one-way ANOVA with Bonferroni post hoc test. Bar, 1 cm.

To further confirm the contribution of glutathione to TIC-like cells survival in 3D gels, we silenced *GCLC*, the catalytic subunit of the rate-limiting enzyme in glutathione biosynthesis, in DG-75 lymphoma cells with shRNA-encoding lentivirus. Among the candidate shRNAs targeting *GCLC*, sh-62, sh-65, and sh-86 demonstrated high knockdown efficacy compared to a non-targeting shRNA (sh-Ctl) in these cells (Fig. 4A). While *GCLC* silencing markedly decreased glutathione levels in DG-75 cells without affecting their viability in liquid culture, it sharply decreased glutathione accumulation and cell viability, and nearly blocked their growth in gels (Fig. 4B–F). To evaluate the role of glutathione in the in vivo tumorigenicity of lymphoma cells, we subcutaneously inoculated DG-75 cells transduced with sh-Ctl or *GCLC*-specific sh-65 and sh-86 in immunodeficient NOD-SCID mice. As expected, control-transduced DG-75 cells readily elicited growth of large tumors in these mice (Fig. 4G). Conversely, *GCLC* silencing markedly impaired tumor growth (Fig. 4G). Similar results were observed in BL-2 lymphoma cells. *GCLC* silencing decreased glutathione accumulation without affecting viability in 2D liquid culture (Fig. S6A–C), but markedly diminished glutathione levels (Fig. S6D), sharply decreased viability (Fig. S6E), and blocked cell growth in 3D gels (Fig. S6F).

Given the potential contribution of GGT to glutathione biosynthesis, we measured its activity in LCLs and lymphoid tumor cells cultured in 2D. We observed markedly higher activity in LCLs relative to tumor cells (Fig. S7A). Since measuring GGT activity in cells grown in 3D is not technically feasible, we cannot rule out that this activity increases under 3D culture conditions. To determine whether GGT contributes to the increased production of glutathione in cells grown in 3D, we treated lymphoid tumor cells with the pharmacological GGT inhibitor GGsTop. This treatment sharply decreased GGT activity (Fig. S7B), modestly decreased glutathione levels (Fig. S7C), and did not substantially reduce the proliferation capacity of lymphoid tumor cells cultured in 2D liquid medium (Fig. S7D). In contrast, GGsTop markedly decreased glutathione levels (Fig. S7E) and nearly blocked colony formation in lymphoid tumor cells cultured in 3D gels (Fig. S7F, G). Collectively, these results emphasize the critical role of glutathione in the growth of TIC-like cells within 3D gels and tumorigenicity.

### Glutathione and ROS dysregulation in tumor cells from lymphoma/leukemia patients

**Fig. 5:**
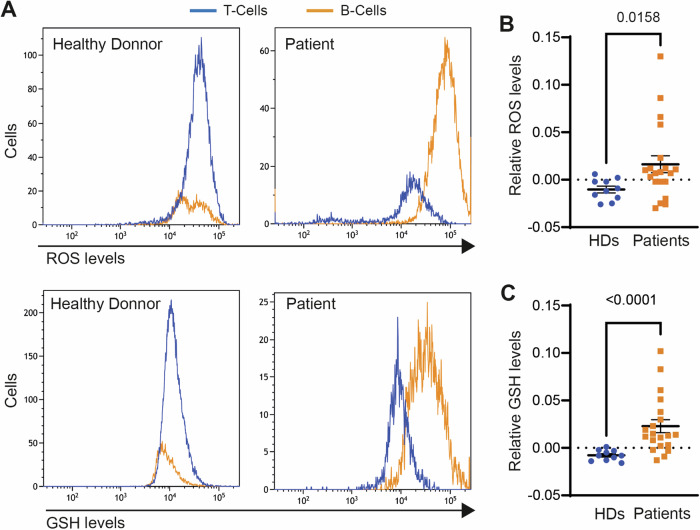
Lymphoid tumor cells contain higher glutathione and ROS levels than non-tumor lymphoid cells. Total blood cells from 10 healthy donors (HD) and 6 acute lymphoblastic B-cell leukemia, 5 chronic lymphoblastic B-cell leukemia, 2 follicular lymphoma, 2 mantle cell lymphoma, 2 splenic marginal zone lymphoma, 1 small lymphocytic lymphoma/chronic lymphocytic leukemia, 1 diffuse large B-cell lymphoma, and 1 hairy-cell leukemia patients were labeled with anti-CD3 and anti-CD19, to identify T and B lymphocytes respectively, and with DCFDA or mBcl as indicators of intracellular ROS and glutathione (GSH), respectively. **A** DCFDA and mBcl staining of a representative HD and patient. The ratio of (**B**) ROS and (**C**) glutathione (GSH) levels in B cells relative to T cells of the same subject is shown. Each data point denotes an individual, and the horizontal bars denote the mean (long bar) and s.e.m. Differences were analyzed by Mann–Whitney test (*p* values are shown).

We aimed to investigate the role of the ROS-glutathione pathway in human cancer by quantifying intracellular ROS and glutathione levels in tumor B cells from B-cell lymphoma/leukemia patients and non-tumor B cells from healthy donors, using non-tumor T lymphocytes as controls. We found that the amount of ROS and glutathione in B lymphocytes relative to T lymphocytes from the same subject was markedly higher in B-cell lymphoma or leukemia patients than in healthy donors (Fig. 5), suggesting that human lymphoma/leukemia cells may increase glutathione production to neutralize the toxic effects of increased ROS production.

### Glutathione overproduction is required for tumor growth in females of a lymphoma mouse model

**Fig. 6:**
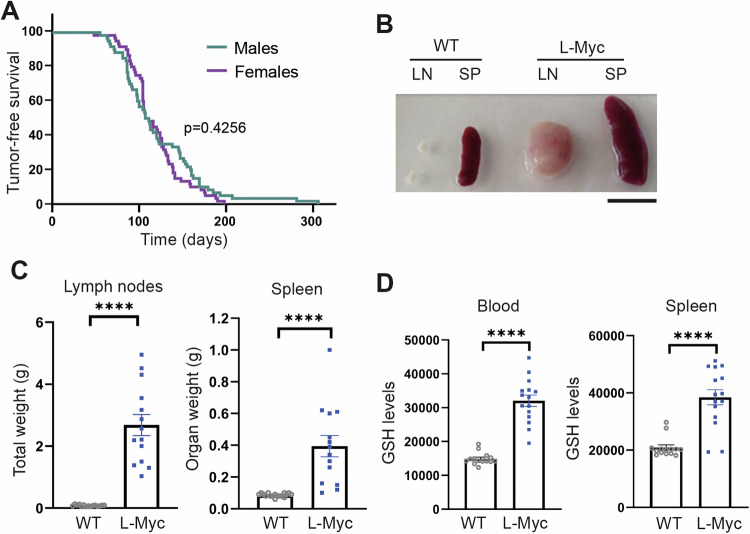
Increased glutathione levels in tumor tissues of a mouse model of B-cell lymphoma. **A** Tumor-free survival curve of 60 male and 60 female λ-Myc mice. Differences were analyzed by Mantel-Cox test; the *p* value is indicated. **B** Representative images and (**C**) spleen weight and aggregated weight of lymph nodes from each of 14 λ-myc mice and 17 WT littermates. Bar, 1 cm. **D** Flow cytometry analysis of mBcl staining (GSH levels) of blood cells and splenocytes from 15 λ-Myc and 14 WT littermate mice. Each data point denotes an individual and data in histograms are presented as mean ± s.e.m. ****p < 0.0001; Student *t* test.

To assess the translational potential of our findings, we investigated the impact of glutathione-mediated ROS neutralization on lymphomagenesis in a preclinical B-cell lymphoma mouse model, C57BL/6N-Tg(Igl-MYC)3Hm (λ-Myc mice) [35]. Lymphoma development in these mice becomes visually evident and palpable around 3 months of age, with >80% of both male and female mice displaying lymphoma signs before 6 months of age (Fig. 6A). Necropsy confirmed splenomegaly and lymph node enlargement (Fig. 6B, C). Flow cytometry analysis of blood cells and splenocytes from λ-Myc mice demonstrated increased intracellular glutathione levels compared to age-matched control littermates (Fig. 6D), indicating a potential requirement for ROS neutralization through glutathione overproduction in lymphomagenesis in vivo.

**Fig. 7:**
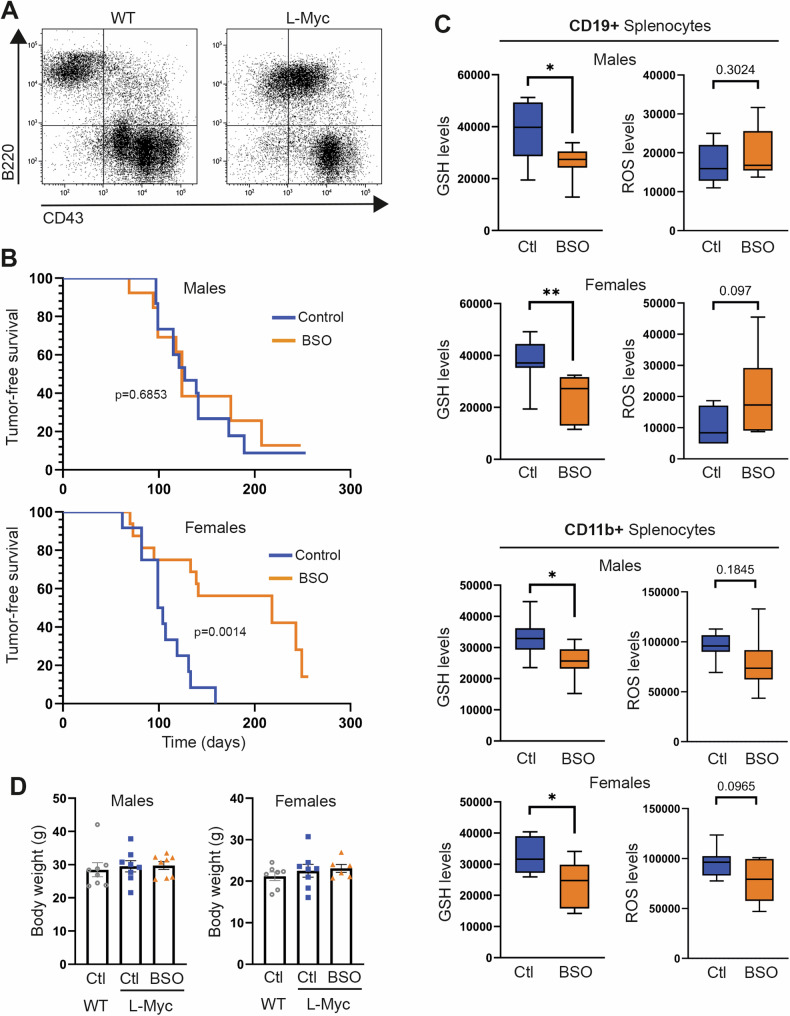
Dimorphic effect of glutathione synthesis inhibition on lymphoma growth in a mouse model of B-cell lymphoma. **A** Representative B220/CD43 double staining of splenocytes from 21-day-old λ-Myc mice. **B** Tumor-free survival curve of 15 male and 12 female untreated (Control) λ-Myc mice and 13 male and 16 female λ-Myc mice treated with BSO in the drinking water (20 mM). Differences were analyzed by Mantel-Cox test (p values are shown). **C** End-of-experiment flow cytometry analysis of mBcl (GSH levels) and DCFDA (ROS levels) staining of CD19+ and CD11b+ splenocytes from 8 male and 7 female untreated (Ctl) λ-Myc mice and 8 male and 7 female λ-Myc mice treated with BSO. Data are presented as box-and-whisker plots, with 75th and 25th percentiles; bars represent maximal and minimal values. *p < 0.05, **p < 0.01, n.s., non-significant by multiple t-test with Holm-Sidak method. **D** End-of-experiment body weight of 8 male and 7 female untreated (Ctl) λ-Myc mice and 8 male and 7 female λ-Myc mice treated with BSO. Each data point denotes an individual and data in histograms are presented as mean ± s.e.m.

To examine this hypothesis and evaluate the therapeutic prospects of pharmacological glutathione synthesis inhibition within the native microenvironment of a lymphoma, we treated λ-Myc mice with BSO. As previously reported [36], an increase in B220^+^/CD43^+^ double-positive tumor cells was observed in the spleen of these mice at 3 weeks of age (Fig. 7A). We therefore initiated treatment with BSO in the drinking water for both male and female λ-Myc mice at P21 and maintained it until 8 months of age, unless humane endpoints were reached. While male mice exhibited minimal changes in tumor formation, treatment of female mice sharply delayed tumor growth (Fig. 7B). Spleen weight and the aggregated weight of lymph nodes were not decreased by the treatment in mice with detectable tumors (Fig. S8), suggesting that BSO may delay tumor initiation rather than tumor expansion. Notably, this treatment markedly decreased glutathione levels in splenocytes of male and female mice (Fig. 7C). Importantly, long-term BSO treatment did not substantially affect mouse body weight (Fig. 7D), suggesting the lack of noticeable toxic effects.

## Discussion

To discover therapeutic strategies that prevent tumor recurrence, we have focused on a unique trait of TICs, their capacity to grow within a 3D gel. Since this characteristic strongly correlates with tumorigenicity in animals [25], we hypothesized that interventions inhibiting TIC growth in 3D gels could also impede tumor development in vivo. Indeed, our previous findings revealed that silencing *CDCA7*, a gene overexpressed in lymphoid tumors, specifically inhibited cell growth in 3D gels, without inhibiting proliferation in 2D conditions, and reduced lymphoma growth in vivo and tumor cell migration and invasion [32, 37]. Our current study shows that inhibiting glutathione synthesis effectively halts the growth of lymphoma cells within 3D gels and hampers lymphomagenesis in a mouse lymphoma model. This challenges the prevailing notion that antioxidants generally benefit health, especially in cancer. We have also shown that primary lymphoma/leukemia cells from this mouse model and B-cell lymphoma/leukemia patients accumulate higher intracellular glutathione levels than non-tumor lymphocytes. This suggests that glutathione overproduction may be indispensable for the survival and proliferation of primary tumor cells, making the inhibition of glutathione production a promising therapeutic avenue for lymphoid tumors.

To the best of our knowledge, the reason non-tumor lymphoid cells do not grow in 3D gels remained unknown. Similar to LCLs, non-tumor epithelial and mesenchymal cells do not grow in these conditions, whereas a minor fraction of their tumor counterparts grows in these gels [26, 27]. This phenomenon has been attributed to the reliance of non-tumor epithelial and mesenchymal cells on a rigid surface for survival [26]. However, the viability of non-tumor lymphoid cells in the circulatory system indicates that anchorage to a rigid surface is not required for their survival. Therefore, the growth disparity in 3D gels may stem from a distinct mechanism. Our findings suggest that some lymphoid cells accumulate in G_0_/G_1_ or G_2_/M, while others experience minimal alteration in cell cycle distribution. In contrast, increased cell death was consistently observed across various lymphoid cell types, including tumor cells, underscoring that, within 3D gels, cell death, rather than cell cycle arrest, impedes lymphoid cell growth.

The inhibition of cell death by the anti-oxidant NAC supports the notion that oxidative stress causes the death of lymphoid cells within 3D gels. Oxygen tension in cell culture incubators (138 mmHg) is higher than in arterial blood (80–100 mmHg) and markedly higher than in tumor hypoxia, where oxygen tension can decrease below 15 mmHg [38]. Although we cannot rule out that the increased production of ROS in 3D culture conditions may not be observed under hypoxia, it should be noted that hypoxia itself induces ROS production in numerous cell types, including tumor cells [39].

Given that GCL or GGT pharmacological inhibition or silencing effectively blocks the growth of lymphoid tumor cells in 3D gels, including TIC-like cells, we propose that high glutathione levels in the minority of tumor cells that thrive in 3D gels counteract ROS-induced cell death. Of note, NAC not only prevents death of non-tumor lymphoid cells in gels but also enables their growth under these conditions. Therefore, caution is advised regarding the administration of high antioxidant doses in diets because an excess might potentially promote overgrowth in antigen-stimulated lymphoid cells. Moreover, as >90% of adults harbor quiescent EBV-transduced B cells [40], high doses of antioxidants might potentially trigger uncontrolled growth in these cells. This hypothesis aligns with reports of EBV presence in various human cancers [41].

While preparing this manuscript for submission, a study based exclusively in in vitro assays was published showing that the addition of 25 µM BSO or the use of tools to delete *GCLC* inhibits the growth of Burkitt lymphoma cell lines under 2D culture conditions [42], seemingly suggesting a comparable susceptibility to glutathione starvation between TICs and differentiated tumor cells. It should be noted that the aforementioned report lack data substantiating *GCLC* deletion or glutathione synthesis inhibition. In contrast, our study confirmed decreased glutathione levels and increased ROS levels through BSO treatment or *GCLC* silencing, and revealed that while the depletion of glutathione did not compromise lymphoma cell survival in 2D culture, it sharply diminished viability in 3D gels. Notably, 3D culture conditions are recognized for its superior ability to predict in vivo efficacy to anticancer drugs than 2D culture in liquid medium [4, 5]. This distinction is critical as >90% of cancer treatments exhibiting preclinical activity fail in human trials, primarily because they are usually tested on cancer cell lines cultured in 2D conditions that inadequately reflect the therapeutic responsiveness of cancers within their native microenvironment [2, 3]. In this regard, our study extends beyond the evaluation of inhibiting glutathione synthesis in 3D culture, demonstrating that neutralizing oxidative stress with glutathione supports lymphoid tumor cells proliferation in vivo. This is evidenced by the inhibition of their growth in immunodeficient mice following lentivirus-mediated *GCLC* silencing. More importantly, pharmacological GCL inhibition impaired lymphomagenesis in a mouse model of B-cell lymphoma. Intriguingly, this effect was observed exclusively in female mice, a result that was unexpected because females generally exhibit a more proficient defense against ROS than males [9]. Although the reasons for this gender-specific outcome remain unknown, our findings underscore the nuanced and complex interplay of glutathione regulation in the context of lymphomagenesis and its potential therapeutic implications.

Other cases of glutathione-dependent sex differences have been reported. For instance, female mice administered BSO exhibited much higher susceptibility to acetaminophen-induced hepatotoxicity than male mice [43], while male mice treated with BSO were much more susceptible to thiabendazole nephrotoxicity than females [44]. Additionally, unlike male birds, female birds treated with BSO during development exhibited longer telomeres, increased growth, and greater body mass than control females in adulthood [45]. Sex differences have also been found in cultured cells; male transformed astrocytes are more dependent on glutathione than female transformed astrocytes to maintain their cellular redox balance [46].

TICs maintain lower intracellular ROS levels than other cancer cells within the tumor, primarily through the accumulation of ROS scavengers like glutathione and the overexpression of Gclm and glutathione synthetase, the enzymes necessary for its synthesis, as shown in breast and liver cancer stem cells [18, 20]. Since most chemotherapeutic agents increase intracellular ROS levels [47], TIC resistance may stem from their high antioxidant content, including glutathione. A potential strategy for achieving complete and relapse-free tumor remission involves combining conventional chemotherapy to target the bulk of tumor cells with drugs that inhibit glutathione synthesis to eliminate TICs. Of note the prolonged treatment with BSO did not substantially affect the weight of treated mice, suggesting limited undesired effects associated with this regimen. Consequently, the suggested combination approach holds the promise of enabling the use of lower doses of conventional chemotherapy, thereby mitigating undesirable toxic effects.

## Methods

### Cell lines and human samples

Human Burkitt lymphoma DG-75 (CRL-2625) and BL-2 (ACC-625), T-cell leukemia Jurkat (Clone E6-1, TIB-152), and HEK-293T (CRL-1573) cell lines were obtained from ATCC (LGC Standards S.L.U., Barcelona, Spain). LCLs JY and X50-7 were from the European Collection of Authenticated Cell Cultures (ECACC 94022533) and Cellosaurus (RRID:CVCL_8277), respectively. DG-75, BL-2, and Jurkat were cultured in RPMI 1640 medium (21875034), whereas HEK-293T were cultured in Dulbecco’s modified Eagle’s medium (DMEM) (11965092), both from ThermoFisher Scientific (Waltham, MA, USA). Both media were supplemented with 10% heat-inactivated fetal bovine serum (FBS) and 2 mM glutamine (25030081), all from ThermoFisher Scientific, plus 100 units/mL penicillin (Laboratorios ERN.S.A., Madrid, Spain; 804443) and 100 μg/mL streptomycin (Reig Jofre S.A., Madrid, Spain; 753483) and were maintained at 37 °C in a humidified incubator with 5% CO_2_. Cell lines were used after receipt or after resuscitation from early stocks at low passage numbers and the identity of lymphoid cell lines was confirmed through STR profiling. All cells were mycoplasma-negative.

Human peripheral blood lymphocytes (PBLs) of healthy donors (HD) were isolated as described [48] from buffy coats, obtained from the Madrid Blood Donor Centre (Madrid, Spain), and then stimulated with 5 µg/ml leucoagglutinin (L4144; Sigma-Aldrich, Madrid, Spain) and cultured in RPMI 1640 medium supplemented with 10% heat-inactivated FBS, penicillin, streptomycin, L-glutamine, and 50 U/mL of human recombinant interleukin 2 (IL2) [49].

Patients included in this study were diagnosed according to WHO and refined consensus criteria [50, 51]. Informed consent was obtained in accordance with the Declaration of Helsinki. Experimental procedures were approved by the Institutional Board of Hospital de La Princesa (PI-802). Cells isolation from freshly donated peripheral blood was done using Ficoll-paque plus density gradient centrifugation (Amersham Biosciences, Little Chalfont, UK). Peripheral blood mononuclear cells (PBMCs) from healthy donors, obtained from peripheral blood or buffy coats, were used as control. Cells were cultured in RPMI-1640 medium supplemented with 10% heat-inactivated FBS, L-glutamine, penicillin, and streptomycin at 37 °C in 5% CO_2_.

### Lentivirus production and cell transduction

Lentiviral particles were produced as previously described [32] employing, psPAX2 and pMD2G-VSVG plasmids [provided by D. Trono (Ecole Polytechnique Federale de Lausanne, Lausanne, Swisse)] and MISSION pLKO.1-puro-based vectors (Sigma-Aldrich, Madrid, Spain,) encoding either a non-targeting shRNA (SHC002) or *GCLC*-targeting shRNAs sh-62 (TRCN0000344862), sh-65 (TRCN0000333565), and sh-86 (TRCN0000048486). Conditioned medium was harvested, filtered through 0.45-μm filters, concentrated by ultracentrifugation (81550 g, 2 h at 4 °C) and stored at −80 °C. DG-75 and BL-2 cell lines were incubated with viral particles in culture medium with 8 μg/ml protamine sulfate during 16 h. Cells were washed to remove viral particles and transduced cells were selected in the presence of 1 μg/ml puromycin (Sigma-Aldrich, P8833) for at least 96 h.

### Western blotting

Cells were harvested and suspended in Total Lysis Buffer (0.125 M Tris-HCl pH 6.8, 4% SDS and 20% glycerol). Cell lysates were boiled for 15 min and protein concentration was determined by the Lowry method. After quantification, β-mercaptoethanol (1:100 v/v) and bromophenol blue powder were added. Protein samples were resolved in 8% SDS-PAGE and transferred to nitrocellulose membranes as described [52]. Membranes were probed with anti-GCLC sc-390811 (200 ng/ml), anti-ACTB sc-69879 (5 ng/ml), and anti-Vinculin sc-73614 (200 ng/ml), both from Santa Cruz Biotechonology (Santa Cruz, CA, USA) followed by goat anti-mouse IgG conjugated to IR-800Dye (926-32210; 1:15,000) from LI-COR Biosciences (Lincoln, Nebraska, USA), and scanned using an Odyssey® Infrared imaging system (Model 9120, LI-COR Biosciences).

### Mouse lymphoma model and tumor xenografts

The previously described mouse model of Burkitt lymphoma C57BL/6N-Tg(IGL-MYC)3Hm (λ-MYC mice) [35] was obtained from the NCI mouse repository (Strain code 01XA7), backcrossed to C57BL/6J for more than 10 generations, and maintained in heterozygosity in a C57BL/6 J background. Mice were monitored weekly for palpable tumors, commencing at an age of 8 weeks. Males and females were treated with 20 mM BSO (19176, Sigma-Aldrich) in the drinking water starting at 21 days of age, immediately after weaning. The experimental endpoint was 250 days of age unless palpable tumors were detected. At endpoint, mice were sacrificed and the spleen and all tumors from each individual mouse were resected and weighed.

Transduced DG-75 cells (2 × 10^6^) in 0.1 ml of phosphate buffer saline (PBS) were injected subcutaneously in the dorsal flanks of 8-to 10-week-old female NOD.CB17-Prkdcscid/J mice (Charles River Laboratories, Wilmington, MA, USA). All mice were inoculated with control cells in one flank and *GCLC*-silenced cells in the opposite flank. Tumor masses were removed after 3 weeks and weighted. All animal procedures were approved by the institutional review board. All animal procedures were approved by the CSIC Ethics Committee (ref. 634/2017 and 1053/2021) and by the Madrid Regional authorities (ref. PROEX 215/17 and 093.7/21), and conformed to EU Directive 2010/63EU and Recommendation 2007/526/EC regarding the protection of animals used for experimental and other scientific purposes, enforced in Spanish law under Real Decreto 1201/2005. Overall mouse health was assessed by daily inspection for signs of discomfort, weight loss, or changes in behaviour, mobility, and feeding or drinking habits.

### Transformation assays in vitro

Agar was prepared in RPMI medium supplemented with 10% heat-inactivated FBS, L-glutamine, penicillin and streptomycin. For colony formation assays, cells (5 × 10^4^) were suspended in 2 mL of 0.33% noble agar (Difco) and laid over 6-well culture plates previously coated with 2 mL of 0.5% noble agar. When appropriate, soft agar was supplemented with 50 µM BSO or 1 mM GGsTop (HY-108467, MedChemExpress; Sollentuna, Sweden). Plates were kept at 37 °C in a humidified incubator in the presence of 5% CO_2_. The number of colonies formed after 3 weeks was counted in triplicate plates.

### Flow cytometry

Cell cycle analysis of cells cultured in liquid medium was performed as described [28] using a FACS Canto II flow cytometer (BD Bioscienes, San Jose, CA, USA). For cell cycle analysis of cells embedded in soft agar, cells (10^5^) were suspended in 1 mL of 0.33% noble agar, seeded in the bottom of 15-mL polypropylene tubes, cooled for 5 min on ice, and then kept at 37 °C in a humidified incubator in the presence of 5% CO_2_ for 24 h–72 h. Agar gels were diluted with 1 mL PBS, melted at 85 °C for 3 min, and centrifuged at 40 °C (5 min at 1800 *g*) to pellet the cells. Cells were washed once with PBS and processed for cell cycle analysis as described [28]. Agarose with control cells was melted and processed, as indicated, immediately after the cooling step.

Cell viability and ROS and glutathione intracellular content of cells cultured in liquid medium were assessed by flow cytometry analysis of cells stained for 30 min in the dark with 0.025 µM calcein-AM (ThermoFisher Scientific, C3099) at room temperature, 50 µM Dichlorodihydrofluorescein-diacetate (DCFDA, ThermoFisher Scientific, D399) at 37 °C, and 10 µM monochlorobimane (ThermoFisher Scientific, M1381MP) at 37 °C, respectively. For analysis of cells embedded in soft agar, cells (10^5^) were suspended in 1 mL of 0.33% noble agar, seeded in the bottom of 15-mL polypropylene tubes, cooled for 5 min on ice, and then kept at 37 °C in a humidified incubator in the presence of 5% CO_2_ for 24 h–72 h. When appropriate, liquid medium and soft agar were supplemented with 50 µM BSO or 1 mM GGsTop. Agar gels were mixed with 1 mL PBS by vigorous pipetting before the addition of calcein-AM, DCFDA, or mBcl to the same concentration as indicated for cells cultured in liquid medium and incubated for 30 min in the dark at the temperature indicated for those cells. A FACS Canto II flow cytometer (BD Biosciences) was used for the analysis employing Kaluza software (Beckman Coulter, Brea, CA, USA).

Splenocytes from λ-MYC mice and wild-type littermates were isolated as previously described [53] and stained with APC-conjugated rat anti-mouse CD45R/B220 (ref 561880) and PE-conjugated rat anti-mouse CD43 (ref 561857) antibodies, both from BD Biosciences, before flow cytometry analysis with a FACS Canto II flow cytometer (BD Biosciences) employing Kaluza software (Beckman Coulter).

### Glutathione and ROS staining of primary T and B cells

Glutathione and ROS staining of human T and B cells was performed on freshly isolated mononuclear cells from peripheral blood or bone marrow samples from 7 control donors and 17 patients. Cryopreserved samples from 3 additional healthy controls and 3 additional patients that could not be analyzed fresh were also included in the study after confirming that ROS and glutathione levels in cryopreserved samples fell within the value range of fresh control and patient samples. Each control sample was analyzed in parallel with one patient’s sample at least. Cells were first stained with 50 µM DCFDA or 10 µM mBcl, as described above, followed by a washing step with PBS and a surface staining of 30 min on ice with combinations of the following monoclonal antibodies: PE-conjugated anti-CD10 (HI10α), PerCP-conjugated anti-CD3 (SK7), APC-conjugated anti-CD19 (SJ25C1), PE-conjugated anti-CD5 (L17F12), PE-conjugated anti-CD25 (PC61), all from BD Biosciences. After another washing step, samples were immediately acquired on a BD FACSCanto™ II with FACSDIVA software (BD).

### GGT activity measurement

GGT activity was measured employing the GGT activity colorimetric assay kit provided by Sigma-Aldrich (MAK089) following manufacturer’s instructions. The OD at 418 nm was measured in a microplate reader FLUOstar® Omega provided by BMG LabTech (Ortenberg, Germany).

### Gene expression analysis

Total RNA was extracted using RNeasy (Qiagen; Venlo, Netherlands) following manufacturer’s instructions. cDNA was prepared from total RNA and used for gene expression by real-time quantitative RT–PCR (qPCR) as described [53] using TaqMan Gene Expression Assays (ThermoFischer Scientific) specific for human *GCLC* (Hs00155249_m1), *OCT4* (Hs00999632_g1), and *ACTB* (Hs01060665_g1). *ACTB* was chosen as a control gene on the basis of its homogeneous expression in non-transduced and control-transduced cells. Each gene expression experiment was performed at least 3 times and calculations were made from measurements of 3 replicates of each sample.

### Statistical analysis

The numbers of animals used are described in the corresponding figure legends. Sample size was chosen empirically based on our previous experience in the calculation of experimental variability. All experiments were carried out with at least three biological replicates. Experimental groups were balanced in terms of animal age, sex, and weight. Animals were caged together and treated in the same way. No randomization was used to allocate animals to experimental groups, and investigators were not blinded to the group allocation during experiments or outcome assessments. GraphPad Prism software 9.4.1. was used for the analysis. Data normality was assessed by the Shapiro-Wilk test, and appropriate tests were chosen according to data distribution. Variance was comparable between groups throughout the manuscript. Differences were analyzed by one-way or two-way analysis of variance (ANOVA) with Bonferroni post-test, Student *t* test, multiple t-test with Holm-Sidak method, Mann–Whitney test, or Mantel-Cox test, as appropriate. Differences were considered significant at *p* < 0.05.

### Supplementary information

Supplementary Figures

Uncropped western blots

## Data Availability

This manuscript does not have data that need to be deposited in a public database. All data analyzed during the current study are available from the corresponding author on reasonable request.
