# Supplementary material for: Glutathione overproduction mediates lymphoma initiating cells survival and has a sex-dependent effect on lymphomagenesis
Source: Cell Death Dis. 2024 Jul 27;15(7):534. doi: 10.1038/s41419-024-06923-z (PMC11283572; doi:10.1038/s41419-024-06923-z)
Supplement: Supplementary file 1 — Supplementary Figures [file 41419_2024_6923_MOESM1_ESM.pdf]

Glutathione overproduction mediates lymphoma initiating cells survival and has a sex-dependent effect on lymphomagenesis

[1,*]Alberto H.-Alcántara, [1,*]Omar Kourani, [2]Ana Marcos-Jiménez, [1]Patricia Martínez-Núñez, [1]Estela Herranz-Martín, [3]Patricia Fuentes, [3]María Luisa Toribio, [2]Cecilia Muñoz-Calleja, [4,5]Teresa Iglesias, and [1,6,#]Miguel R. Campanero

Supplementary Figures & legends

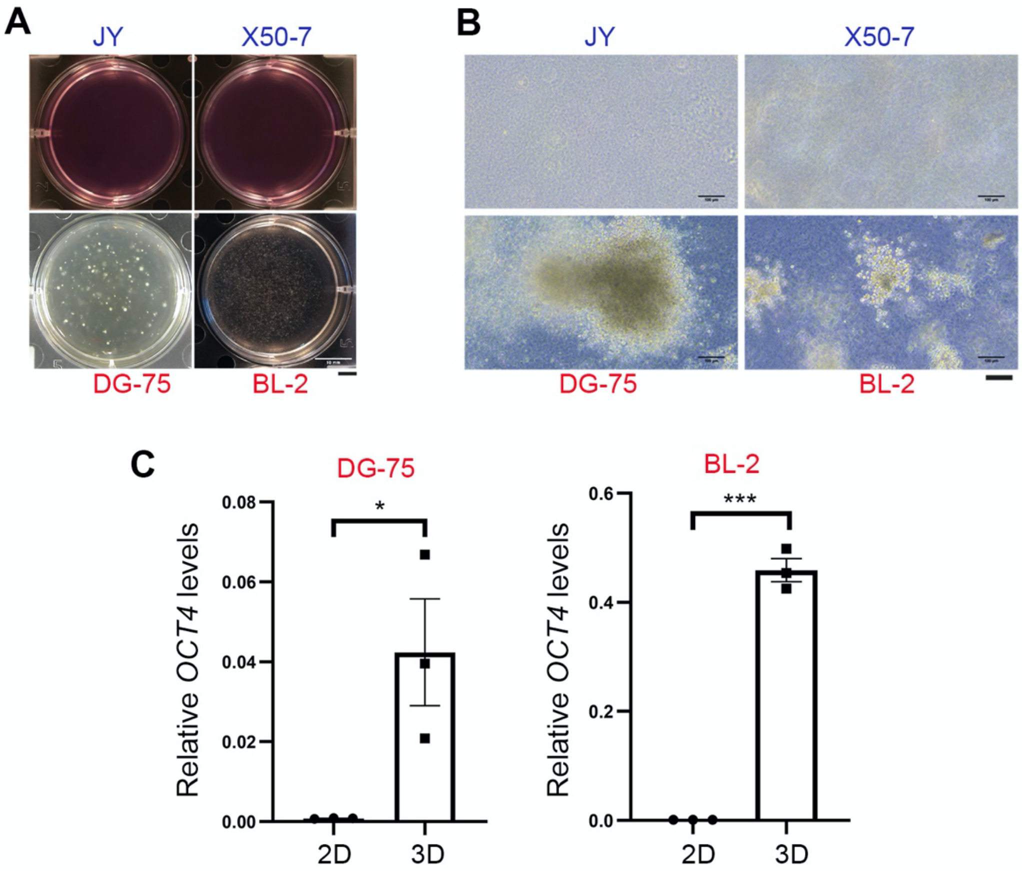

Supplementary Figure 1. Increased *OCT4* expression in lymphoma cells grown in soft gels. (**A**) Macroscopic and (**B**) microscopic images of representative wells containing LCLs (JY and X50-7) or lymphoma cells (DG-75 and BL-2) grown within 3D soft-agar hydrogels for 21 days. Bars, 10 mm (A) and 100 µm (B). (**C**) *OCT4* mRNA expression, as assessed by RT-qPCR, in extracts from DG-75 and BL-2 cells grown in 3D hydrogels for 21 days (n = 3 independent experiments). mRNA amounts were normalized to those of *ACTB*. Data are means ± s.e.m. *P<0.05, ***P<0.001, by paired t-test.

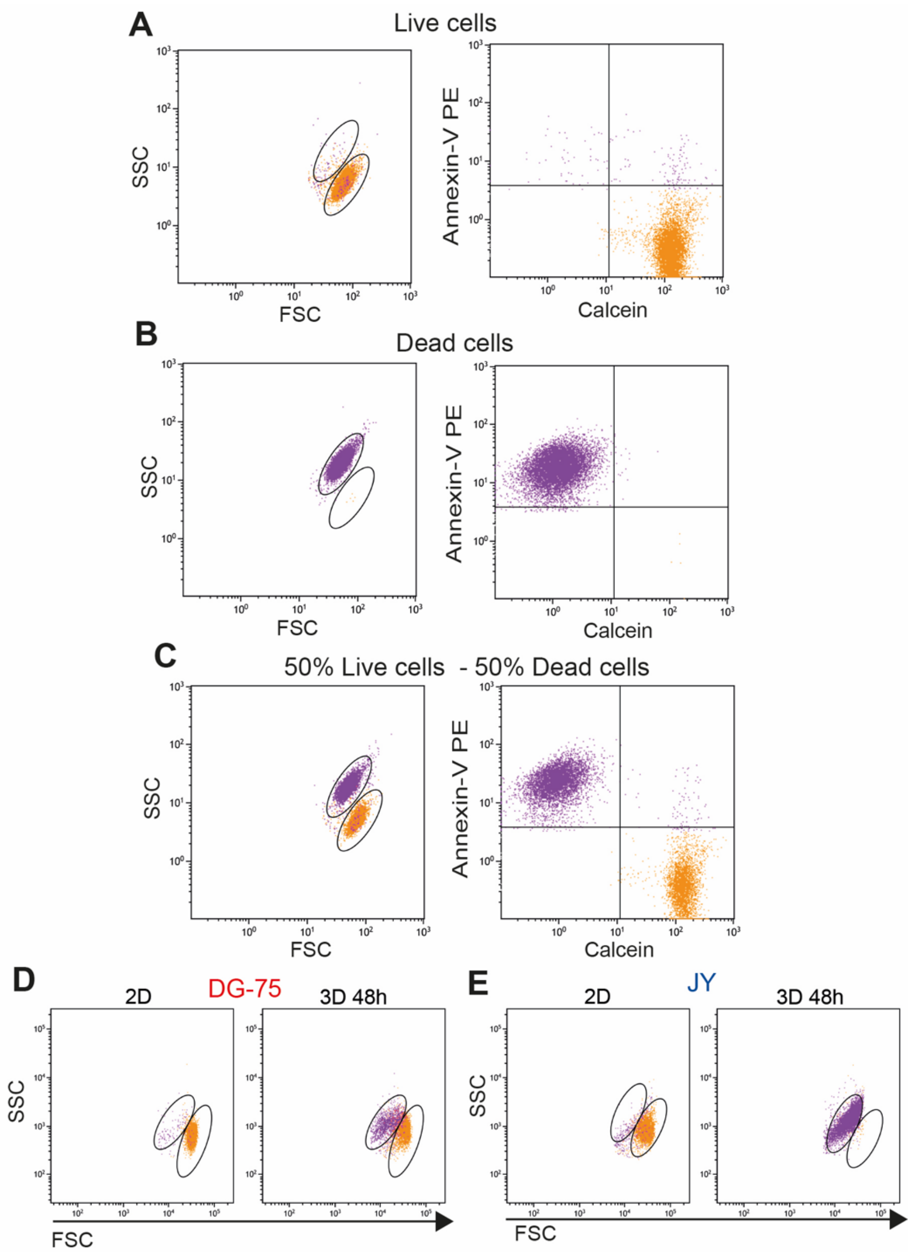

Supplementary Figure 2. Cell size and granularity analysis by flow cytometry distinguishes live and dead cells. Flow cytometry analysis of DG-75 cells incubated at 65ºC for 15 minutes or left untreated and stained with Calcein-AM and phycoerythrin-conjugated annexin V. Forward scatter (FSC) and side scatter (SSC) dot plots (left panels) and Annexin-V and Calcein dot plots (right panels) of (**A**) control DG-75 cells, (**B**) DG-75 cells incubated at 65ºC for 15 minutes, and (**C**) a 50% mixture of control and heated DG-75 cells. FSC/SSC dot plots of (**D**) DG-75 and (**E**) JY cells cultured in 2D liquid medium or within a 3D gel for 48h.

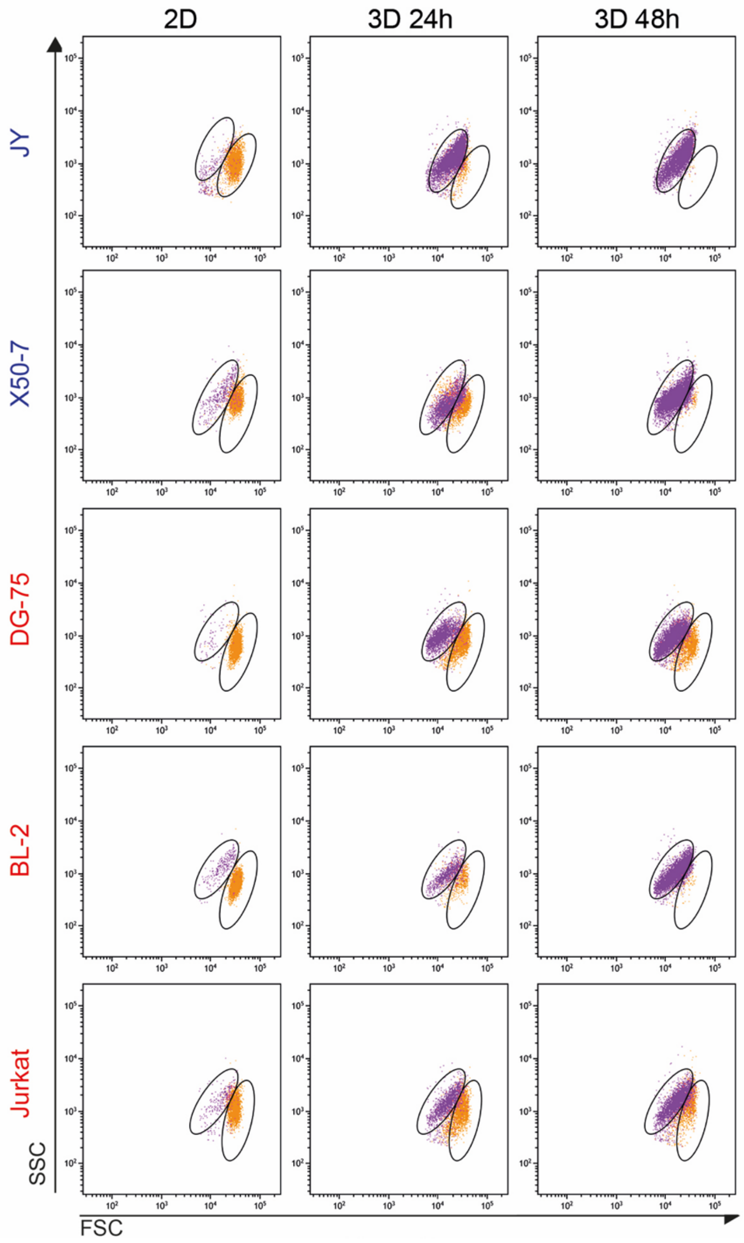

Supplementary Figure 3. Cell culture in soft-agar gels induces cell death. Flow cytometry analysis of cell size (FSC) and granularity (SSC) of the indicated cell lines cultured in 2D liquid medium or within a 3D soft agar gel (3D) for 24h or 48h. Live (orange) and dead (purple) cells populations are indicated.

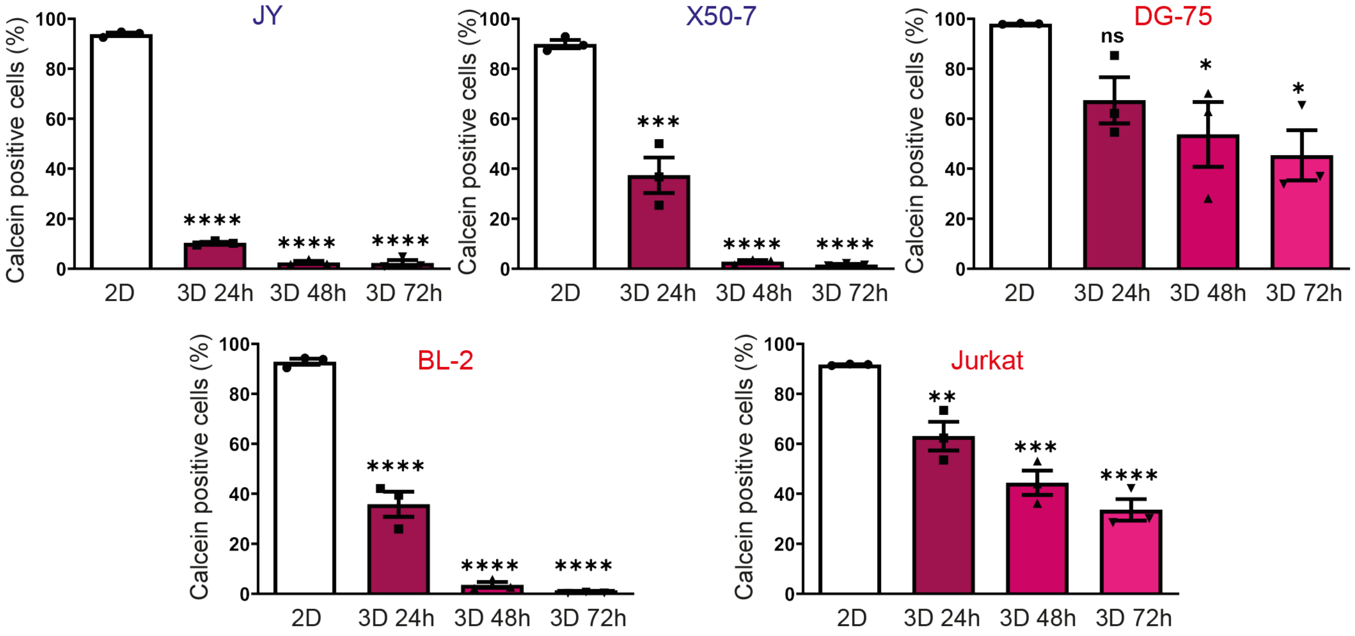

Supplementary Figure 4. Cell culture in soft gels decreases cell viability. The rate of living cells was determined by flow cytometry analysis of calcein staining of JY, X50-7, DG-75, BL-2, and Jurkat cells cultured either in 2D liquid medium or within a 3D soft-agar hydrogel for 24h, 48h, or 72h. Each data point denotes the value from an independent experiment and data in histograms are presented as mean + s.e.m. *p<0.05, **p<0.01, ***p<0.001, ****p<0.0001, ns, non-significant; one-way ANOVA with Bonferroni post hoc test.

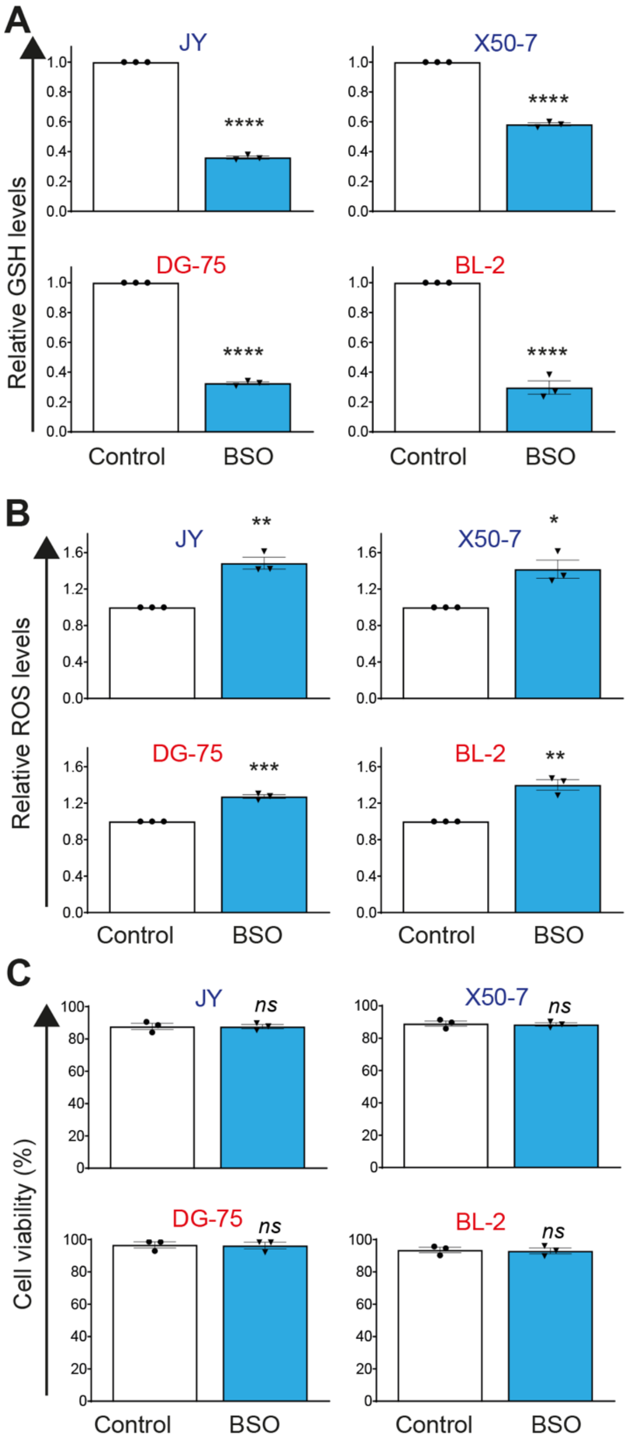

Supplementary Figure 5. Glutathione synthesis inhibition increases oxidative stress but does not induce lethality in lymphoid cells grown in 2D liquid medium. The indicated cells were cultured for 24h in 2D liquid medium in the absence (Control) or the presence of 50 μM BSO. Flow cytometry analysis of the staining of these cells with (**A**) mBcl or (**B**) DCFDA. (**C**) Rate of living cells as determined by flow cytometry analysis of cell size and granularity. Each data point denotes the value from an independent experiment and data in histograms are presented as mean ± s.e.m. *p<0.05, **p<0.01, ***p<0.001, ****p<0.0001, n.s., non-significant, Student t-test.

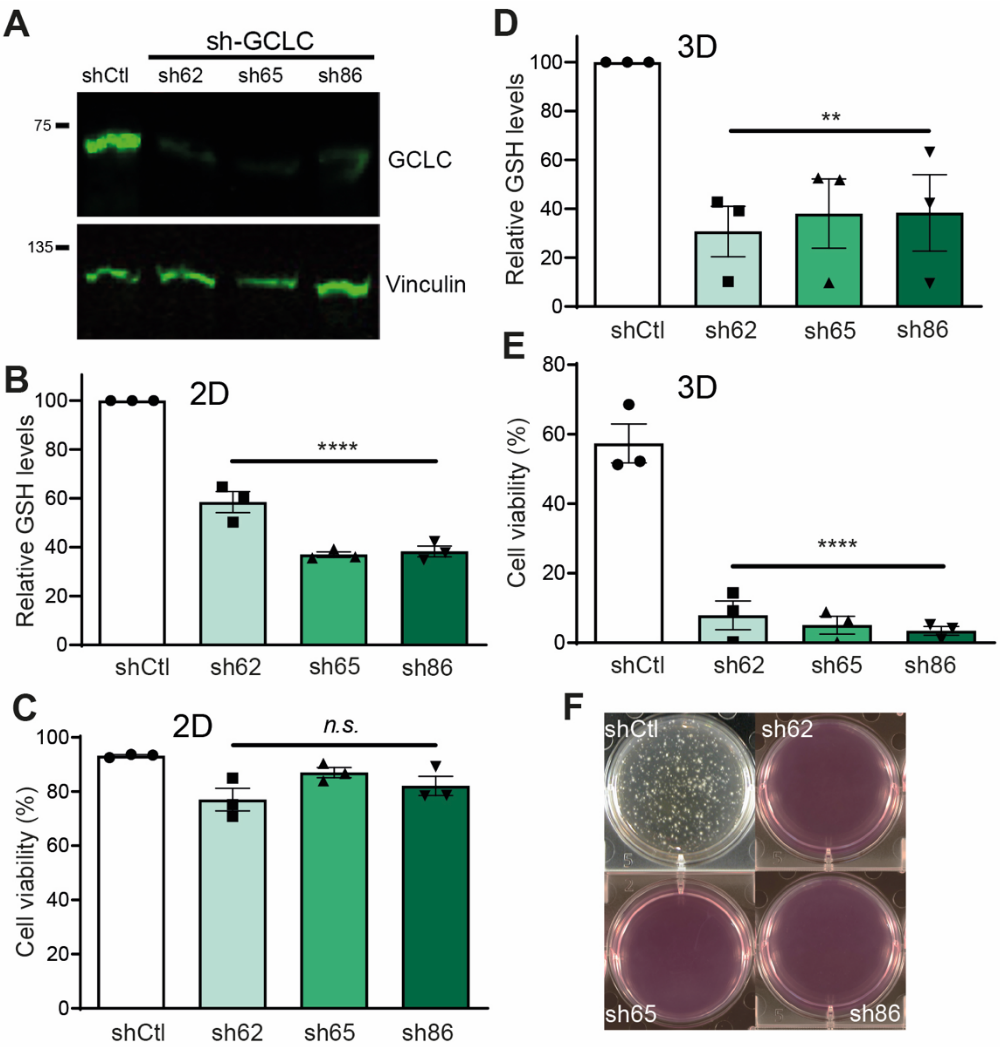

Supplementary Figure 6. *GCLC* silencing impairs BL-2 lymphoma cells survival and growth in soft gels. BL-2 cells were transduced with lentivirus encoding a puromycin-inactivating protein and either a control shRNA (shCtl) or the *GCLC*-specific shRNAs sh-62, sh-65, or sh-86. Transduced cells were selected by culture for >5 days in 2D liquid medium containing 2 µg/ml puromycin and then further cultured (**A-C**) in 2D liquid medium or (**D-F**) within 3D soft-agar hydrogels. (A) Representative GCLC and Vinculin (loading control) immunoblot analysis of BL-2 cells transduced as indicated (n = 3 independent cell batches per group). Uncropped images of these immunoblots are shown in a Supplementary File. Flow cytometry analysis of (B,D) mBcl staining and (C,E) cell size and granularity of cells transduced as indicated after 24h of culture in 2D liquid medium (B,C) or within a 3D gel (D,E). Each data point denotes the value from an independent experiment and data in histograms are presented as mean ± s.e.m. **p<0.01, ****p<0.0001, *n.s.*, non-significant, one-way ANOVA with Bonferroni post hoc test. (F) Images of representative wells containing the indicated transduced BL-2 cells grown within 3D gels for 21 days.

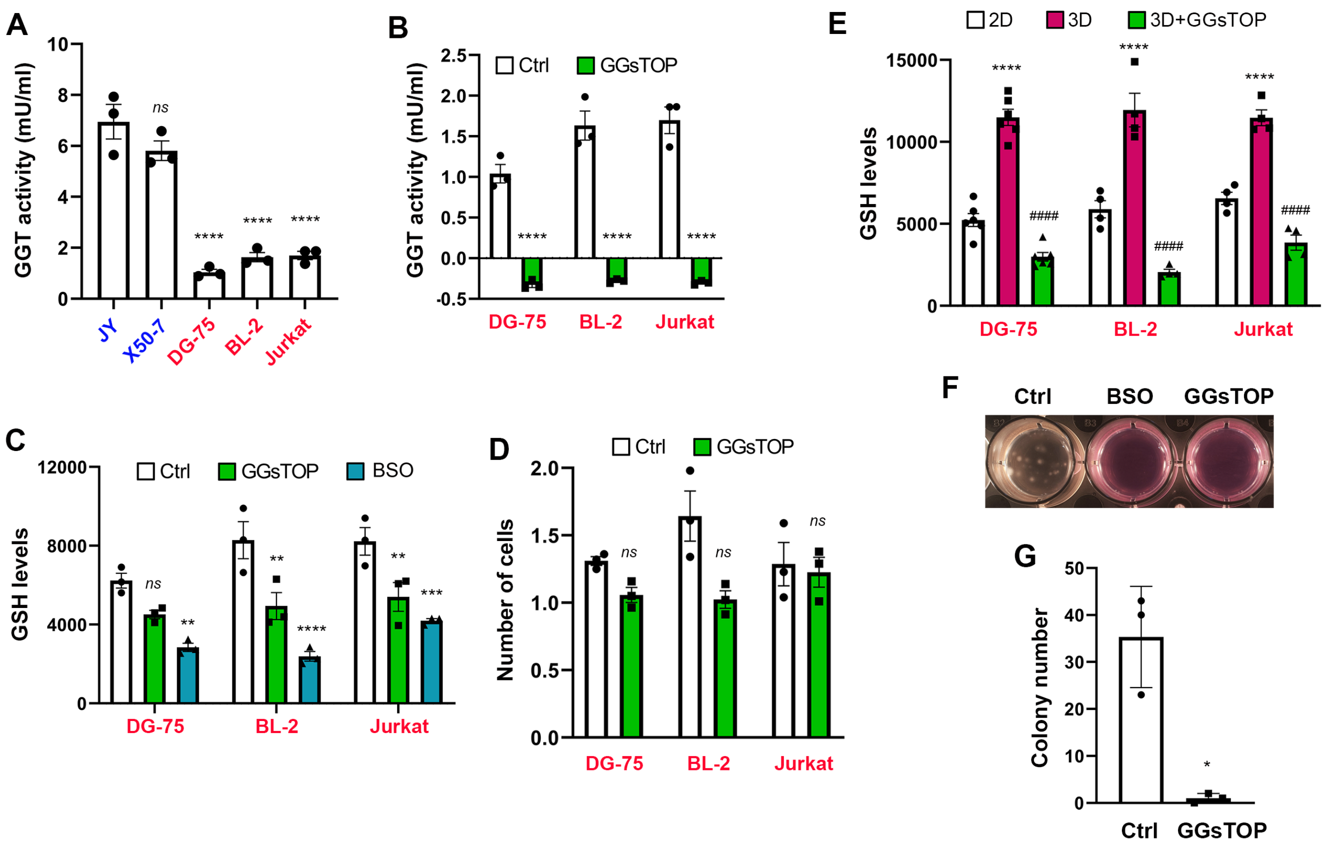

Supplementary Figure 7. GGT inhibition impairs glutathione accumulation and survival in lymphoid tumor cells cultured in 3D. (**A**) Basal GGT activity determination in the indicated cells cultured in liquid medium and (**B**) liquid medium in the presence of 1 mM GGsTOP for 72h or water, used as control (Ctrl). (A) *ns*, not significant; ****P<0.0001 vs JY; one-way ANOVA with Bonferroni post hoc test. (B) ****P<0.0001 vs Ctrl; two-way ANOVA with Bonferroni post hoc test. (**C**) Flow cytometry analysis of glutathione (GSH) staining with mBcl in the indicated cells cultured in liquid medium (2D) in the absence (Ctrl) or presence of 1 mM GGsTOP or 50 μM BSO for 24h. Each data point denotes the median fluorescence intensity from an independent experiment and histograms are presented as mean ± s.e.m. of 3 independent experiments. *ns*, not significant; **P<0.01, ***P<0.001, ****P<0.0001 vs Ctrl; two-way ANOVA with Bonferroni post hoc test. (**D**) DG-75, BL-2, and Jurkat cells (125 000 cells) were cultured in liquid medium for 72h in the absence or presence of 1 mM GGsTOP. Each data point denotes the average number of cells in a triplicate culture at the end of the experiment and histograms are presented as mean ± s.e.m. of 3 independent experiments. *ns*, not significant; multiple t-test Holm-Sidak method. (**E**) Flow cytometry analysis of glutathione (GSH) staining with mBcl in the indicated cells cultured in liquid medium (2D) or 3D gels in the absence (3D) or presence of 1 mM GGsTOP (3D+GGsTOP). Each data point denotes the median fluorescence intensity from an independent experiment and data in histograms are presented as mean ± s.e.m. ****P<0.0001 vs 2D; ####P<0.0001 vs 3D; two-way ANOVA with Bonferroni post hoc test. (**F**) Images of representative wells containing the indicated cells grown in 3D in the absence (Ctrl) or presence of 50 μM BSO or 1 mM GGsTOP for 21 days and (**G**) number of colonies per well. Each data point denotes the value from an independent experiment and data in histograms are presented as mean ± s.e.m. *p<0.05; paired Student t-test.

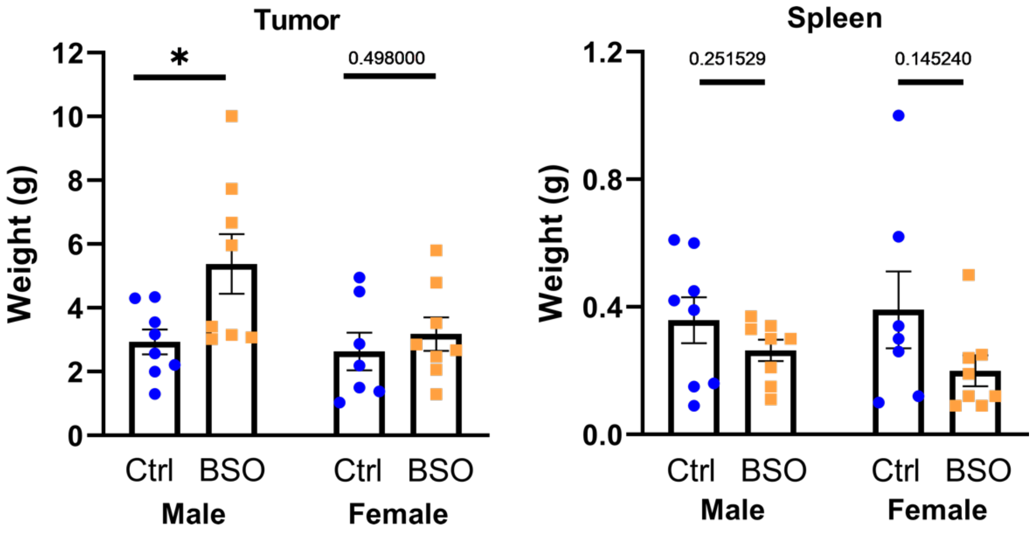

Supplementary Figure 8. Spleen and tumor weight in mice with detectable tumors. Spleen weight and aggregated weight of lymph nodes from untreated (Ctrl) and BSO-treated λ-Myc mice with tumors detected by palpation. Each data point denotes an individual and data in histograms are presented as mean + s.e.m. Differences were analyzed by multiple t-test with Holm-sidak method (p values are shown; *P<0.05).
