# Supplementary material for: Glutathione overproduction mediates lymphoma initiating cells survival and has a sex-dependent effect on lymphomagenesis
Source: Cell Death Dis. 2024 Jul 27;15(7):534. doi: 10.1038/s41419-024-06923-z (PMC11283572; doi:10.1038/s41419-024-06923-z)
Supplement: Supplementary file 2 — Uncropped western blots [file 41419_2024_6923_MOESM2_ESM.pdf]

UNCROPPED IMMUNOBLOTS

Figure 4A

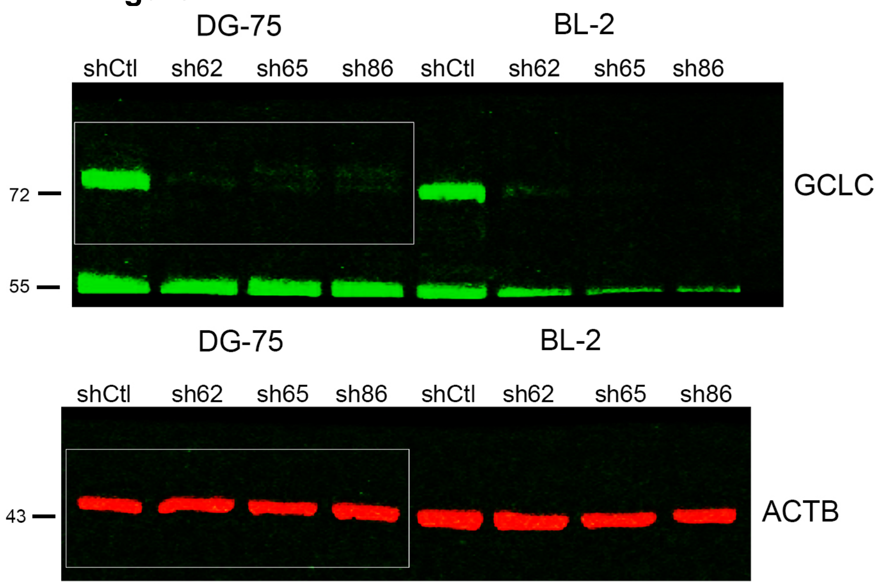

Supplementary Figure 6A

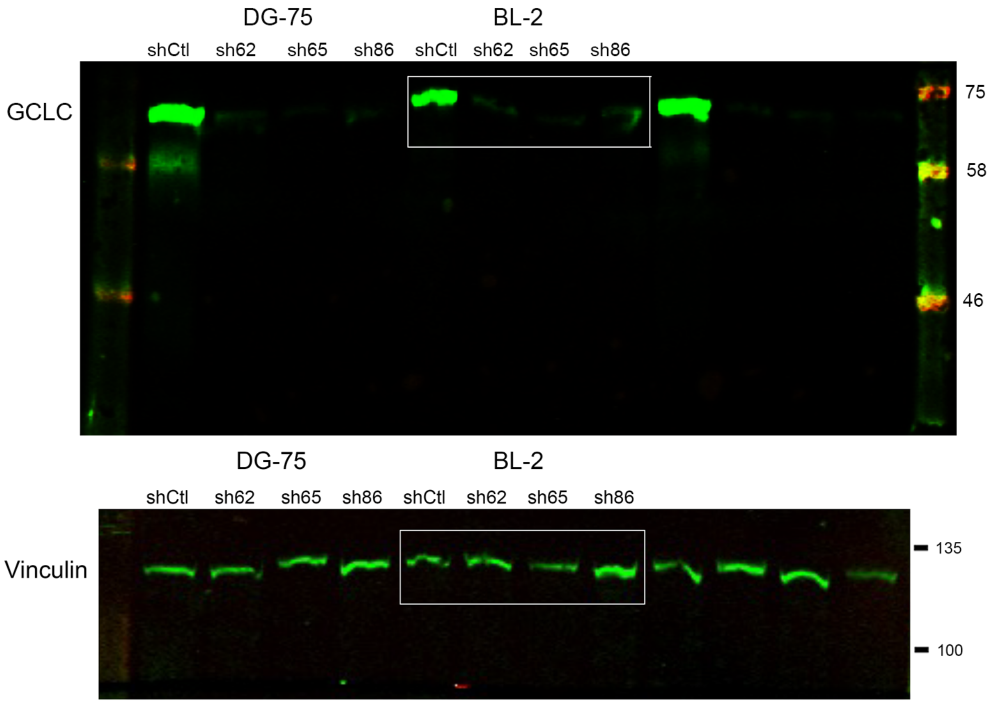

Supplementary Figure 9. Representative uncropped images of immunoblots shown in Figure 4A and Supplementary Figure 6A.
